# Reduced Stability and Increased Dynamics in the Human Proliferating Cell Nuclear Antigen (PCNA) Relative to the Yeast Homolog

**DOI:** 10.1371/journal.pone.0016600

**Published:** 2011-02-18

**Authors:** Alfredo De Biasio, Ricardo Sánchez, Jesús Prieto, Maider Villate, Ramón Campos-Olivas, Francisco J. Blanco

**Affiliations:** 1 Structural Biology Unit, CIC bioGUNE, Derio, Spain; 2 Structural and Computational Biology Programme, Centro Nacional de Investigaciones Oncológicas (CNIO), Madrid, Spain; 3 IKERBASQUE, Basque Foundation for Science, Bilbao, Spain; Griffith University, Australia

## Abstract

Proliferating Cell Nuclear Antigen (PCNA) is an essential factor for DNA replication and repair. PCNA forms a toroidal, ring shaped structure of 90 kDa by the symmetric association of three identical monomers. The ring encircles the DNA and acts as a platform where polymerases and other proteins dock to carry out different DNA metabolic processes. The amino acid sequence of human PCNA is 35% identical to the yeast homolog, and the two proteins have the same 3D crystal structure. In this report, we give evidence that the budding yeast (sc) and human (h) PCNAs have highly similar structures in solution but differ substantially in their stability and dynamics. hPCNA is less resistant to chemical and thermal denaturation and displays lower cooperativity of unfolding as compared to scPCNA. Solvent exchange rates measurements show that the slowest exchanging backbone amides are at the β-sheet, in the structure core, and not at the helices, which line the central channel. However, all the backbone amides of hPCNA exchange fast, becoming undetectable within hours, while the signals from the core amides of scPCNA persist for longer times. The high dynamics of the α-helices, which face the DNA in the PCNA-loaded form, is likely to have functional implications for the sliding of the PCNA ring on the DNA since a large hole with a flexible wall facilitates the establishment of protein-DNA interactions that are transient and easily broken. The increased dynamics of hPCNA relative to scPCNA may allow it to acquire multiple induced conformations upon binding to its substrates enlarging its binding diversity.

## Introduction

**Figure 1:**
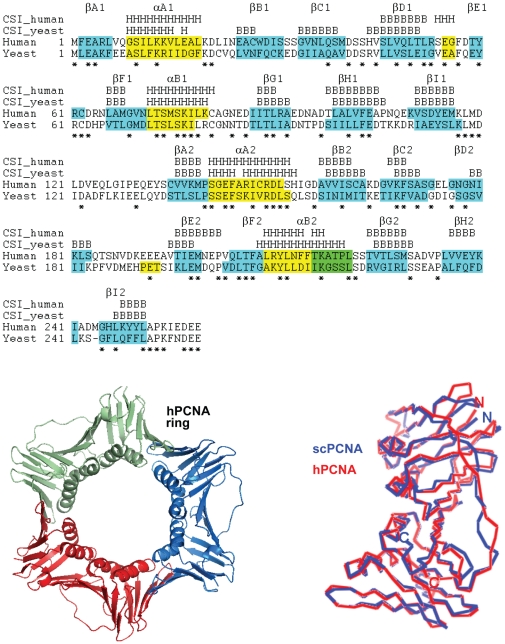
Structure of the PCNA sliding clamps. Amino acid sequence alignment of human and yeast PCNA monomers. The conserved residues are indicated with an asterisk. The secondary structure elements within the two topologically identical domains were assigned from the crystal structures (PDB entries 1VYM and 1PLQ), sequentially labeled βA1, βB1, etc. (first domain) and βA2, βB2, etc. (second domain) and highlighted (light blue for β strands and yellow for α helices, in green are indicated residues annotated in a 3/10 helix conformation). The secondary structure of yeast and human PCNA molecules identified in solution using the Chemical Shift Index [44] obtained from the NMR chemical shifts is indicated above the sequences. H stands for α-helix and B for β-sheet. This index relies on the consensus of only three measurements at each position (only the ^13^C^α^, ^13^C^β^, and ^13^C' were assigned, but not the ^1^H^α^), and therefore there is a large number of residues for which an overall consensus could not be reached. At the bottom-left the superposition of the C^α^ trace of human PCNA (red) and yeast PCNA (blue) subunits is shown. At the bottom-right, a ribbon representation of the human PCNA trimeric ring is shown. Structures were prepared using PyMol (http://www.pymol.org).

DNA replication, a fundamental event for the maintenance and transfer of the hereditary information through generations, requires a complex molecular apparatus called replisome [1]. A central component of the replisome is represented by the DNA sliding clamps. These consist of multimeric, toroidal-shaped structures with pseudo-six fold symmetry that encircle the DNA duplex and act as processivity factors during replication by tethering the replicative polymerases to the genomic template. All kingdoms of life retain functionally and structurally related sliding clamps that differ in the multimeric association of monomeric subunits [2]. The bacterial clamp (DNA polymerase III β subunit) is formed by homo-dimeric association of two protomers, each one with three topologically similar domains [3], [4]. In contrast, the archaeal and eukaryotic clamps (PCNAs) assemble into trimeric rings in which each 29 kDa protomer contains two similar domains with 2 β-sheets, 2 α-helices and a long interdomain-connecting loop (IDCL) (Figure 1) [4], [5]. The PCNA monomers are arranged in a head-to-tail fashion, forming a ring with an internal diameter of approximately 34 Å, large enough to accommodate the DNA duplex. The ring is formed by an outer layer of 6 β-sheets and an inner layer of 12 α-helices that line the central channel. Although both human and scPCNA have negative net charges, the distribution of positively charged residues on the helices lining the inner surface of the ring creates a positive electrostatic potential in the central channel, so that the negatively charged DNA duplex can sit and pass through it with little repulsion [4]. The PCNA rings are stable in solution [6] and need to be opened to be loaded onto the DNA [7]. The clamp loader (replication factor C) mediates the assembly of PCNA onto DNA in an ATP dependent process [8].

In addition to the replicative function, PCNA directs other important cellular processes through the interaction of a host of DNA-processing proteins and cell cycle regulators [9]. These interacting partners bind to a region located on the interdomain-connecting loop of each PCNA monomer [10] via their PCNA-interacting protein motifs (PIPs).

The structure of DNA sliding clamps has been highly conserved during evolution, which is consistent with their pivotal role in DNA replication and repair. The shape, size and internal architecture of the prokaryotic DNA polymerase III β subunit are maintained in eukaryotic PCNAs despite a very low level of sequence identity (∼10%) and a different oligomeric state [4]. Within eukaryotes, the sequence and structure conservation is obviously more prominent, with the budding yeast homolog of PCNA sharing a 35% of amino acid identity with the human one (Fig. 1). The question we pose in this work is whether the virtual structural identity of yeast and human PCNAs is mirrored by similar thermodynamic stability and internal dynamics. We present a detailed comparative analysis showing that the yeast homolog of PCNA is more resistant to chemical and thermal denaturation and that it is characterized by higher cooperativity of unfolding. By making use of our previously assigned backbone NMR resonances of both yeast and human PCNA trimers [11], [12], we performed an amide hydrogen/deuterium solvent exchange analysis in order to assay and compare the local backbone dynamics of the two proteins. Our NMR data show that yeast PCNA is more rigid than its human homolog on the time-scale of this experiment. We discuss these results in the context of protein evolution and propose a functional role for the decreasing stability and increasing flexibility in evolving PCNA.

## Results

### scPCNA and hPCNA sequence and structure comparison

The human and yeast PCNA homologs share 35% of sequence identity and their crystal structures are highly superimposable over the elements of secondary structure (with a RMSD of 1.0 Å for the C^α^ atoms, Fig. 1). Differences exist regarding the overall content of different types of amino acids: the yeast protein contains larger and smaller percentages of charged and uncharged polar residues, respectively, as compared to the human homolog (Table 1). The same trend is observed between the thermophilic *P. furiosus* homolog (pfPCNA) and the yeast homolog, in agreement with the general trend observed for thermophilic proteins [13].

**Table 1:** Structural analysis of the PCNA intermonomer interfaces and overall amino acid compositions.

Homolog	Number of backbone-backboneHydrogen Bonds^1^	Number ofBackbone-side chainHydrogen Bonds^1^	Number ofSalt Bridges^1^	ASA (Å^2^) 1	Percentage of charged residues (DEKRH)	Percentage of Polar Uncharged residues (GSTNQYC)	Percentage of Hydrophobic residues (LMIVWPAF)
hPCNA	8	4	1	709	26	32	42
scPCNA	8	1	1	654	29	28	44
pfPCNA	5	9	6	718	33	22	45

1 The analysis was carried out using the PDB*e*PISA tool for exploration of macromolecular interfaces. The PDB entries used were 1PLQ for scPCNA, 1VYM for hPCNA and 1GE8 for pfPCNA.

The PCNA trimer is formed through intermolecular main chain amide-to-carbonyl hydrogen bonds between the anti-parallel β-strands BI1 and BD2. The analysis of the crystallographic structures of human and yeast PCNA identified 8 amide-to-carbonyl hydrogen bonds formed in the interface of both homologs (Table 1). In addition, 4 hydrogen bonds involving side chains are detected in the interface of hPCNA, while only one in scPCNA. In both homologs, one salt bridge is present at the interface: between Lys110 and Glu143, and between Arg110 and Asp150 in hPCNA and scPCNA, respectively. The guanidinium group of Arg110 of scPCNA also binds Asp86 in the same subunit of scPCNA. The buried Accessible Surface Area (ASA) per monomer at the interface is 708 and 654 Å^2^ for the human and yeast homolog, respectively. The same analysis carried out on the structure of pfPCNA homolog reveals a total of 14 hydrogen bonds, 6 ionic pairs and an ASA of 719 Å^2^ at the interface.

### Structure of human and yeast PCNA molecules in solution

The far-UV circular dichroism spectra of scPCNA and hPCNA are consistent with proteins with mixed α/β/coil secondary structures (Figure S1 and Supporting Note S1). We have analyzed the backbone NMR chemical shifts of the two proteins, and found that the same helices and sheets as in the crystal are present in solution, as shown in Fig. 1. During the course of the assignment of the ^1^H-^15^N NMR correlation spectra of the two proteins [11], [12], a number of amide-amide NOEs were identified and found to match those expected from the short distances measured in the corresponding crystal structures (). Analytical ultracentrifugation experiments at 100 µM show that the populations of both hPCNA and scPCNA monomers in equilibrium with their trimeric rings is less than 3% (Fig. S3). This measurement is in agreement with previous reports showing that hPCNA is predominantly trimeric in solution at concentrations above 50 nM [14] and is stable over a 25–45°C temperature range [6].

The existence of loosely bound dimers of trimers has been reported for both endogenous and recombinant hPCNA after cross-linking with formaldehyde [15]. We have further analyzed the oligomeric state of the two homologs using gel filtration coupled to multi-angle light scattering (MALS), which is very sensitive to large aggregates because of the non-linear dependency of scattering with the particle size. In our hands, the trimeric form is the predominant one in both human and yeast PCNA at 10 or 100 µM concentrations, with a minor population (less than 4%) of species with the molecular weight of a dimer of trimers (Fig. S4).

The structure of both PCNA molecules changes at high temperatures, but in a complex way, possibly due to simultaneous secondary, tertiary and quaternary structure rearrangements (Fig. S5 and S6).

### Folding-unfolding equilibrium measurements

**Figure 2:**
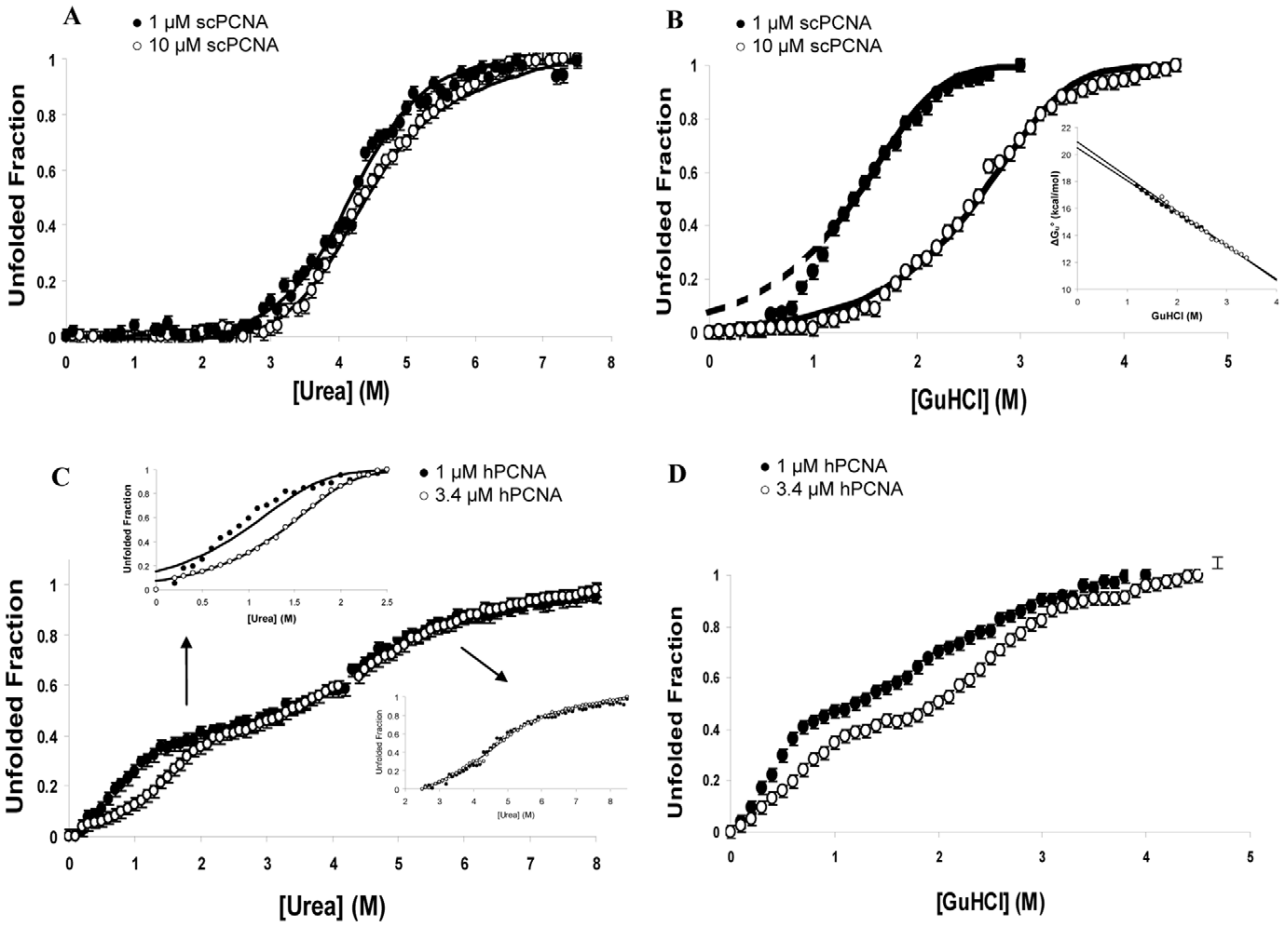
Chemical denaturation of PCNA. Denaturation of PCNA molecules was monitored by recording the change in the CD signal at 222 nm, which was then used to compute the fraction of unfolded protein. Open and filled circles represent the higher and lower concentrated samples, respectively. Circles correspond to experimental data and solid lines represent the fittings to theoretical models. The estimated thermodynamics parameters are assembled in Table 2. (A) Urea-induced denaturation of 1 µM and 10 µM scPCNA. (B) Urea-induced denaturation of 1 or 3.4 µM hPCNA. The insets show the fitting of the curve between 0 and 2.5 M urea (left) and between 2.4 and 8.5 M urea (right) (C) GuHCl-induced denaturation of 1 and 10 µM scPCNA. The values of 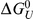 in the transition region (from 1 M to 3.5 M GuHCl) calculated using equation 7 (derived from equation 3) are plotted in the inset together with the result of a linear regression fitting (solid line) that yields the free energy at zero denaturant concentration. Note that fitting to equation 3 results in a free energy per mol of trimer, while in table 2 the reported figure has been normalized per mol of monomer and therefore is three times smaller. Because the lower branch of the theoretical curve derived from the extrapolated value of 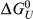 poorly fits the experimental data, it is shown as a dashed line (D) GuHCl-induced denaturation of 1 and 3.4 µM hPCNA. All measurements were done at 35°C in 20 mM sodium phosphate buffer pH 7.0, 150 mM NaCl.



The folding-unfolding curves of the two homologs induced by urea or GuHCl are strikingly different (Fig. 2). Urea denaturation of scPCNA follows an apparent two-state process (Fig. 2A) with a mid point transition of 4.3 M urea at both 1 and 10 µM protein concentration, almost concentration independent. These observations can be interpreted in two ways: either scPCNA trimer dissociates prior to the transition zone of the curve, and only the equilibrium between folded monomer and unfolded monomer is observed, or the trimer unfolds without dissociating and what is observed is the transition between the folded trimer to an unfolded trimeric species. Both mechanisms can be described by unimolecular reactions in which the equilibrium populations of folded and unfolded protein is independent of the total concentration:

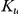


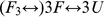
(1)

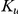


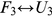
(2)where *F* indicates the protein native state, *U* the unfolded state and *K_u_* is the equilibrium constant of unfolding. The curves were fitted according to a two-state model and the estimated standard free energies of unfolding at zero denaturant concentration are the same at the two concentrations (Table 2).

**Table 2:** Thermodynamic parameters for scPCNA and hPCNA denaturation by Urea and GuHCl.

	[scPCNA](µM)	[hPCNA](µM)	MidpointDenaturantConcentration(M)	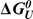 (kcal⋅mol^−1^) a	*m*(kcal⋅mol^−1^⋅M^−1^)
Urea	1	-	4.3	4.8±0.3^b^	1.1±0.1
	10	-	4.4	4.9±0.2^b^	1.2±0.1
0–2.5 M	-	1	0.8	6.5±0.1^c^	2.6±0.2
	-	3.4	1.4	6.50±0.01^c^	2.85±0.02
2.5–8.8 M	-	1	4.6	2.9±0.5^d^	1.4±0.2
	-	3.4	4.7	2.9±0.4^d^	1.1±0.1
GuHCl	1	-	1.4	6.97±0.04^e^	2.56±0.05
	10	-	2.6	6.80±0.04^e^	2.42±0.04

a Standard free energy of unfolding at 35°C extrapolated to zero denaturant concentration (See materials and methods). For simplicity all these values are expressed per mol of monomer (unless otherwise specified, see next note).

b The two possible mechanisms (data fitting to equations 1 or 2) yield the same numeric result (but per mol of unfolded monomer or per mol of unfolded trimer, respectively). If any of these models is correct, the value would not include the contribution of dissociation.

c From equation 4.

d From equation 5.

e From equation 3.

The folding-unfolding of scPCNA induced by GuHCl (Fig. 2B) also shows a two-state behavior but strongly dependent on the protein concentration. The data can be described by a concerted reaction of dissociation and unfolding [16]:
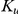


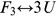
(3)


If unfolding occurs via this mechanism, the estimated equilibrium constants (and the free energies of unfolding) should be the same in experiments performed at different protein concentrations. The curves at protein concentrations of 1 and 10 µM were fitted according to equation 3 and the values of the fitted thermodynamic parameters are summarized in Table 2. At 1 µM protein concentration the estimation of the pre-unfolding baseline is not optimal and the fitting is poor for the initial points. Still, the very similar values of the free energies of unfolding calculated at the two protein concentrations supports a two-state folding-unfolding mechanism of scPCNA in GuHCl.

The folding-unfolding of hPCNA in the presence of urea or GuHCl shows a biphasic behavior consistent with a partially unfolded intermediate state which is significantly populated in the transition zone (Fig. 2C and 2D). The urea denaturation curves recorded at two protein concentrations differ only in the region 0–2.5 M urea (Fig. 2C). This dependence on protein concentration suggests that hPCNA unfolding is coupled to the dissociation of the ring. Assuming that the formation of the initial partially unfolded-dissociated species and its subsequent unfolding are two independent processes, the curve can be split into two parts and analyzed separately. With this assumption, the equilibrium in the range 0–2.5 M urea was fitted to a two-state model according to the following reaction:
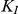


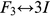
(4)where *I* is the intermediate state and *K_I_* is the equilibrium constant. The calculated free energies of formation of the intermediate at the two protein concentrations are the same within the experimental error, larger at low protein concentration because of the uncertainty in the fitting of the baseline at low urea concentrations. The second part of the curve (between 2.5 and 8.5 M urea) was fitted to a two-state unimolecular reaction:
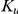


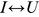
(5)


As shown in table 2, the extrapolated free energies of unfolding are very similar at the two concentrations.

The folding-unfolding curve of hPCNA in GuHCl (Fig. 2D) is also biphasic but dependent on protein concentration throughout the whole range of denaturant concentrations. This curve could not be fitted to a simple model.

### Backbone amide hydrogen exchange with the solvent

**Figure 3:**
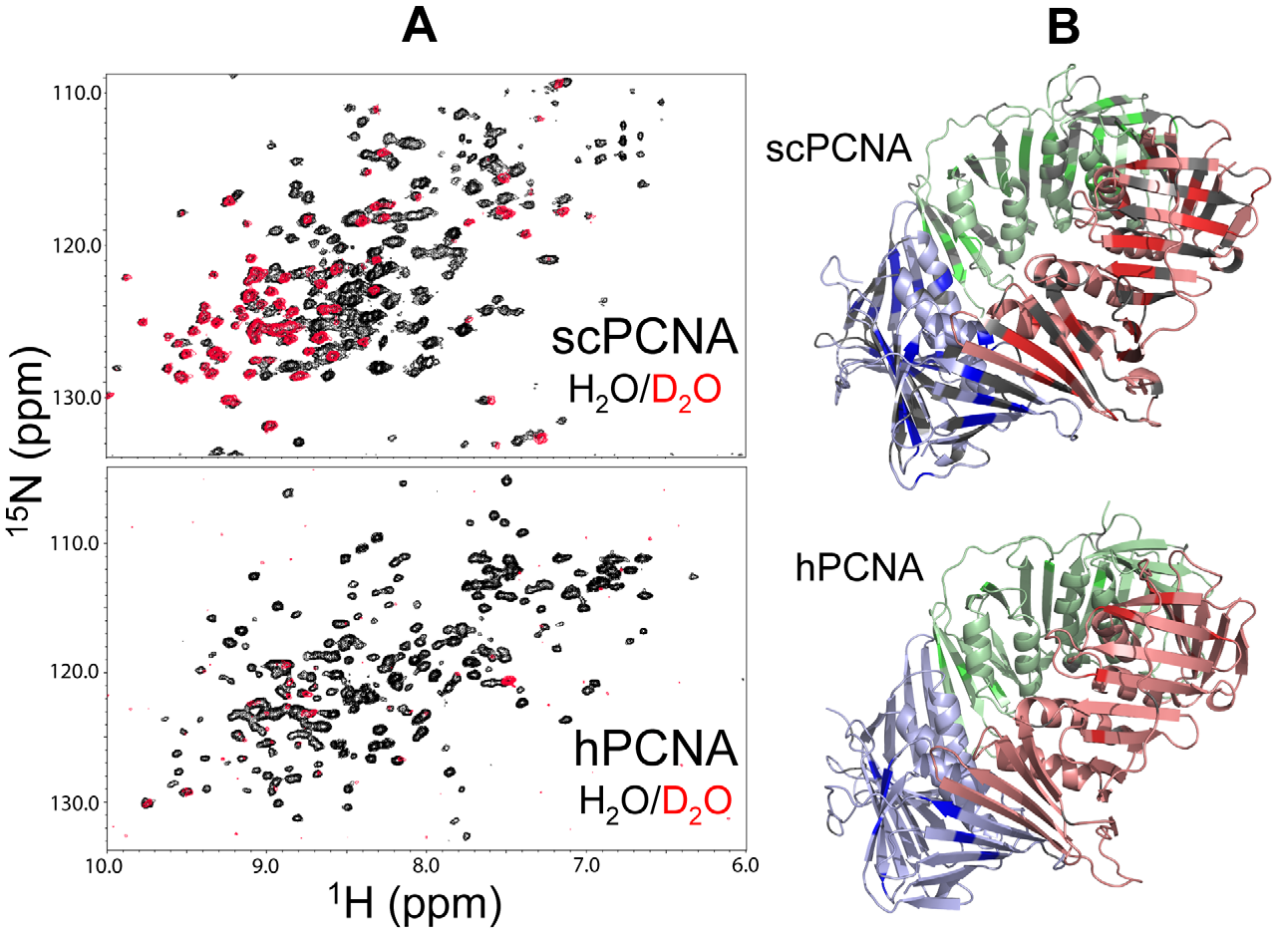
Solvent exchange protection of PCNA backbone amide protons. (A) Overlay of ^1^H-^15^N correlation spectra of scPCNA (TROSY-type, upper panel) and hPCNA (HSQC-type, lower panel) in buffered 5% D_2_O (black) or immediately after placing them in buffered ∼100% D_2_O (red). (B) The ribbon diagrams represent the scPCNA (upper) and hPCNA (lower) trimeric rings. The three protomers in both diagrams are colored in light red, blue and green. The residues in scPCNA for which a protection factor could be estimated or those in hPCNA whose signal could be detected immediately after D_2_O exchange, are highlighted in a brighter shade of the corresponding color in the protomer. The residues in scPCNA for which a reliable protection factor could not be obtained (due to low signal intensity or signal overlap) are colored in grey. Prolines in both structures are also colored in grey.

The dynamics of local unfolding was monitored by NMR measurements of the proton/deuterium exchange of the backbone amides with the solvent (see Supporting Note S1). A prominent difference in the global rate of exchange of the amidic protons of scPCNA versus hPCNA can be appreciated by looking at the reference ^1^H-^15^N correlation spectra (in buffered H_2_O) and those recorded immediately (∼1 hr) after D_2_O exchange (Fig. 3). Most of the backbone amide resonances are visible in the reference spectra of both proteins; however, while 90 out of the 257 amide resonances of scPCNA are still clearly observed after ∼1 h in D_2_O, only 15 of the 260 cross-peaks of hPCNA could be seen under similar conditions. Moreover, all hPCNA cross-peaks are exchanged beyond detection after a few hours. The complete assignment of the backbone amide resonances of both proteins [11], [12] allows for a residue-specific analysis of the proton exchange rates (Fig. S7, S8 and Table S1). Reliable exchange constants (*k_ex_*) and corresponding protection factors could be measured for 35 of the 90 scPCNA residues that were still visible immediately after buffer exchange (Fig. 3 and Supporting Table S1), while for the remaining ones this could not be possible due to either signal overlap or a too rapid decay. Considering the sensitivity and the initial time and duration of the first NMR experiment, an upper limit of *k_ex_* (0.6 and 1.2 s^−1^ for scPCNA and hPCNA, respectively) was estimated for those protons which exchanged too fast for a reliable constant to be measured.

## Discussion

The two PCNA homologs have virtually identical crystal structures and the NMR chemical shifts and NOEs show that the solution structures are also the same, although we cannot exclude the existence of conformational differences in the coil regions when the proteins are in solution. No major differences in the backbone hydrogen bond and salt bridge patterns, or in the accessible surface areas at the intermonomer interfaces are observed between the two homolog, although the larger number of hydrogen bonds in the hPCNA points to a more stable trimer. Interestingly, the relatively high number of salt bridges observed at the interface of pfPCNA suggest that this may be the dominating factor for the extreme thermostability displayed by this homolog [17].

A correlation seems to exist between protein stability and the enrichment/depletion of specific classes of amino acids [18]. Although this is a statistical rule that may not apply to individual proteins the trend observed in the relative amino acid composition of the human, yeast and the thermophilic PCNA proteins predicts that the human homolog is less stable than the yeast one. The thermal denaturation of both PCNA molecules is complex but hPCNA starts losing secondary structure at a temperature about 15°C lower than scPCNA. Therefore hPCNA is less resistant than to thermal denaturation than scPCNA.

The chemically induced folding-unfolding equilibria of scPCNA and hPCNA are also complex, as expected for a macromolecular assembly of 90 kDa. The folding of scPCNA resembles a two-state transition while the folding of the human homolog proceeds through at least one partially unfolded intermediate. The fact that the denaturant-induced formation of the hPCNA intermediate depends on the protein concentration suggests that this intermediate is dissociated.

We have tried to estimate the free energies of unfolding of the two proteins by fitting the data to the simplest models that could reasonably explain the experimental measurements, as summarized in Table 2. The standard free energy of unfolding of scPCNA derived from the GuHCl induced folding-unfolding equilibria at 35°C, and assuming a two state folding mechanism (equation 3) is 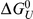 = 6.9 kcal⋅mol^−1^. The corresponding GuHCl curve for hPCNA cannot be fitted to any simple model preventing a quantitative comparison of the stability of the two homologs. The use of urea to observe the folding-unfolding equilibria resulted in curves that could be fitted for both scPCNA (equations 1 or 2, with sequential or simultaneous dissociation/unfolding) and hPCNA (equations 4 and 5, three state mechanism) yielding a difference (hPCNA minus scPCNA) in the global free energy of unfolding of 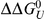 = 6.5+2.9−4.8 = 4.6 kcal⋅mol^−1^. This number means that hPCNA is more stable than scPCNA, but it may be an overestimation if the free energy of scPCNA unfolding does not include the contribution from dissociation (both of the two mechanism behind equations 1 and 2 fit well the data, but only if equation 1 applies is the dissociation energy included in the unfolding free energy). If we assume that scPCNA in urea folds-unfolds according to equation 1 (unfolding after dissociation), and that in GuHCl does so according to equation 3 (with concomitant unfolding and dissociation), the standard free energy of dissociation at 35°C would be 7.0−4.8 = 2.2 kcal⋅mol^−1^ (and 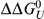 would remain 4.6 kcal⋅mol^−1^). However, if equation 2 (unfolding without dissociation) applies in urea, the standard free energy of scPCNA dissociation would be 7.0−4.8/3 = 5.3 kcal⋅mol^−1^ (the division by 3 is here necessary to subtract two quantities expressed in the same units, kcal per mol of *monomer* units; see table 2), and 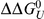 = 6.5+2.9−4.8−5.3 = −0.7 kcal⋅mol^−1^ (with scPCNA being more stable that hPCNA). Since hydrogen bonds, salt bridges and buried accessible surface areas of the intermonomer interfaces are not very different between the two PCNA rings, it is probable that both unfold into dissociated monomers, that equation 1 applies to scPCNA unfolding in urea, and that hPCNA is globally more stable than scPCNA. This would be consistent with the larger number of hydrogen bonds at the interface in the structure of hPCNA (Table 1). A rigorous quantitative comparison of their unfolding free energies needs the unambiguous identification of the folding-unfolding mechanisms of the two PCNA molecules. However, we can conclude that while the two homologs become close to fully unfolded at similar urea concentrations (about 6.5 M) the population of the partially unfolded hPCNA is maximal at urea concentrations (about 2.5 M) where scPCNA is still predominantly in its folded state (dissociated or not). Therefore, even though the value of 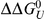 may be positive, hPCNA is less resistant than scPCNA to chemical denaturation.

Because there are not many detailed studied on the stability of oligomeric proteins it is interesting to compare our results on PCNA with those of other proteins. The value of 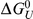 = 6.9 kcal⋅mol^−1^measured for scPCNA at 35°C is similar to the value of 6.2 kcal⋅mol^−1^ measured at 37°C for the α+β Villin 14T protein, which is monomeric, about half sized, and unfolds with a two-state mechanism [19]. In contrast, the α+β trimerization domain of T7 fibritin (ten times smaller than PCNA, but trimerizing via β-hairpin structures in each monomer that resemble the intermonomer antiparallel β-strand pairing in PCNA rings) displays a two-state unfolding transition from trimer to unfolded monomers with a 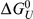 = 21.3 kcal⋅mol^−1^ at 25°C [20]. The three times larger difference could be due to the lower temperature, to a different mechanism of scPCNA unfolding or to both. The study of the folding kinetics of scPCNA could provide information about the similarities or differences in its folding mechanism with the smaller protein. While we estimate a dissociation energy between 2.2 and 5.3 kcal⋅mol^−1^ at 25°C smaller than the unfolding free energy, a ten fold larger free energy for dissociation than for unfolding has been measured for the heptameric human chaperonin10 protein [21].

Eukaryotic PCNA rings were shown to be stable trimers (*K_d_* ∼21 nM, [14]) over the range of temperatures 25–47°C [6]. However, a difference between the two homologs concerning the equilibrium of dissociation of the trimer may exist, and could have relevant implications in the step of ring opening in the multi-step mechanism of loading of the clamp onto the DNA [8]. Indeed we could detect the presence of a small population of hPCNA monomers in equilibrium with the trimeric rings at concentrations not very far from those used in the denaturation experiments (Fig. S3, S4). The only known structure of a monomeric PCNA protein is from the archeon *S. sulfataricus*, whose PCNA ring is heterotrimeric [22]. The structure of one of the isolated monomers is maintained in the trimer (pairwise C^α^ RMSD ∼1.0 Å) with the differences concentrated in the regions that contact the other two molecules of the ring. However this does not necessarily means that the monomers of PCNA proteins of other species are stably folded in isolation.

The solvent exchange protection factor of an amide proton can be interpreted in terms of its participation in a hydrogen bond or solvent inaccessibility or both. Exchange is thus facilitated by local unfolding or structural rearrangement events. In the PCNA structure only 44% of the amide protons are involved in hydrogen bonds, most of them located in the regular secondary structure elements, and the distribution is similar in human and yeast PCNA. The lower protection observed in the human homolog indicates that the local structural stability is lower, or less energy is required to populate the exchange-competent, higher energy forms. This result is consistent with the thermodynamics data.

Since the hydrogen-exchange assay provides insight into the presence of locally unfolded conformations under native conditions in exchange with the folded ones, smaller exchange rates correspond to slower local backbone dynamics [23], [24], [25], [26]. Therefore, our measurements on the homologous PCNA molecules support a more dynamic state of hPCNA as compared to scPCNA. Most of the exchange-resistant amides are located in the β-sheet core of the monomers, especially in their central strands, and are involved in hydrogen bonds. The amides located in the loops of scPCNA show limited protection and only two exchange-resistant residues (^78^Ile and ^79^Leu) are located in the four helices of the subunit. Thus, the backbone of the helices is locally more dynamic than the backbone of the central β-sheet core. As for hPCNA, all the backbone amide signals that were detected in the first NMR spectrum after D_2_O buffer exchange decayed so fast that a reliable exchange rate constant could be not obtained. Still these few more resistant amide protons are located in the β-sheet (Fig. 3) suggesting that the differential dynamics between the helices and the β-sheet is common to both PCNA molecules.

The increased dynamics of the α-helices, which face the DNA duplex in the PCNA-loaded form, relative to the outer β-sheet layer has functional implications as well concerning the sliding of DNA through the hole of the ring. Because the clamp moves on the DNA, the established interactions are transient and therefore easily broken [27], a situation facilitated by a large hole with a flexible wall.

From an evolutionary perspective, DNA sliding clamps represent a highly successful molecular machine whose structure and function have been preserved in all branches of life despite profound differences in the amino acid sequence and oligomeric states [2]. The mutations acquired by the human homolog of PCNA relative to the yeast homolog preserve the existing three dimensional structure but reduce its stability and folding cooperativity and increase the local backbone dynamics. These changes in the structural features of PCNA may represent an evolutionary strategy for buffering destabilizing mutations, the vast majority and whose accumulation leads to a loss of functional protein. According to this hypothesis, named gradient robustness adaptation [28], the selective pressure would favor mutations that give proteins increased structural flexibility coupled to lower stability, since in well-packed, highly connected polypeptide chains sequence changes are likely to be more disruptive. In addition to functioning as an adaptive control of stability loss, the enhanced flexibility acquired by PCNA, a protein involved in a complex network of interactions [10], may represent a selective advantage in relation to interaction with other proteins. The acquired plasticity may constitute a gain of function as it would allow multiple induced conformations upon binding to the substrates, improving the binding diversity [29], [30]. Most known interactors bind to the interdomain connecting loop of PCNA [2] through the so-called PIP (PCNA interacting protein) box, and the emerging picture reveals a flexible interaction that requires more than one mode of interaction [31]. The interaction of human PCNA with a peptide derived from tumor suppressor protein p21WAF1 causes a rigidification of the flexible linker strand (residues 121–132) and the C-terminal tail (residues 251–261) of PCNA, as inferred from the change in the crystallographic B-factors [32]. Studies on the interaction between an archeal homolog of PCNA and FEN-1 (Flap-endonuclease 1) [33] reveal that the binding is established by the ordering of flexible C-terminal regions of PCNA and FEN-1 which creates an intermolecular β-sheet interface (although this was not observed in the human PCNA-FEN-1 complex [34]). Flexibility seems to be a requirement on the PCNA ligand side as well: in the *Sulfolobus solfataricus* DNA ligase-PCNA complex, the interaction occurs via a PIP motif (Q-X-X-I/L/M-X-X-Y/F-Y/F, present in many PCNA-interacting proteins [10]) located in a flexible loop of the ligase [31], while the N-terminus of *Saccharomyces cerevisiae* Msh6, a protein of the Mut-s complex that recognizes sites of DNA mismatch, was shown to be an unstructured tether to PCNA [35].

As organisms evolve, the more complex interactome regulating DNA replication, repair, and cell cycle control may require a higher tolerance by PCNA to accommodate the binding of a larger variety of PCNA ligands, presenting more diverse PIP sequences. This higher tolerance can indeed be mediated by the enhanced flexibility and lower stability of the human PCNA over its yeast homolog characterized here. The lower stability and increased flexibility may be important for a finer control of hPCNA degradation [2]. Recently the function of yeast PCNA was found to be highly affected by mutations that modify its affinity for several of its other protein partners, with higher affinities causing deficiencies in DNA replication and DNA repair more severe than binding abolishment [36]. This surprising observation shows that biological processes can be robust and fragile, depending on the set of mutations examined. It would be interesting to perform a similar analysis with the more plastic human PCNA.

## Materials and Methods

### Protein expression and purification

The human and yeast PCNA proteins were produced and purified as previously described [11], [12]. Briefly, the genes were subcloned with an N-terminal poly-histidine tag and a *PreScission* protease cleavage site. Protein samples with natural isotopic abundance or enriched in ^2^H and ^15^N were obtained by expression in *E. coli* cells grown in the appropriate culture media. The proteins were purified from the soluble fraction by several chromatography steps, and contained the extra sequence GlySerHis- at the N-terminus after proteolysis and removal of the affinity tag. Stock solutions (in 137 mM NaCl, 2.7 mM KCl, 10 mM sodium phosphate, 2 mM potassium phosphate pH 7.0) were flash frozen in liquid nitrogen and stored at −80°C. Protein concentration of the samples was measure by absorbance at 280 nm using the extinction coefficient calculated from the amino acid composition. The concentrations of PCNA indicated in this work always refer to the corresponding concentration of monomers.

### Chemically induced folding-unfolding measurements and analysis

The urea and GuHCl denaturation curves were measured at 35°C with a Jasco-815 polarimeter using an automatic titrator (Jasco ATS 429) with 0.1 M increments.The CD signal at 222 nm was used to monitor the unfolding of scPCNA and hPCNA at different concentrations of urea or GuHCl. Each denaturant addition to the protein solution (1 and 10 µM scPCNA or 1 and 3.4 µM hPCNA in 10 mM sodium phosphate, 150 mM NaCl, pH 7.0) was made from a protein stock solution of the same concentration but containing 9 M Urea or 7.4 M GuHCl, so that the concentration of the protein in the measuring quartz cuvette (1 cm path length) was kept constant. A period of 30 seconds after denaturant addition was allowed for equilibration of the folding-unfolding reaction in the sample before recording the CD signal. Urea concentration was estimated by dry-powder weighing and confirmed by measuring the refractive index. The buffered GuHCl stock was prepared from commercial 8M GuHCl solution in water (Sigma). The experimental urea denaturation curve of scPCNA and the curve of hPCNA in the range 2.5–8.8 M urea were fitted to two-state equilibria (*F ↔U* in the case of scPCNA, or *I ↔U* in the case of hPCNA) using the following equations [37]:
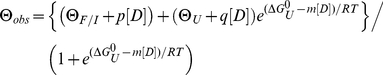
(6)where *Θ_obs_* is the observed CD signal at 222 nm at a given denaturant concentration, *Θ_F/I_* is the signal of the folded protein (*F*) for scPCNA or the hPCNA intermediate (*I*), *Θ_U_* is the signal of the unfolded state (*U*), *p* and *q* are the slopes of pre- and post-unfolding baselines, respectively, [D] is the concentration of denaturant, 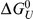 is the standard free energy of unfolding at zero denaturant concentration [38], and *m* is a measure of the dependence of the unfolding free energy on the concentration of the denaturant, *T* is the temperature in kelvin degrees (308 K), and *R* is the ideal gas constant (1.985 cal⋅K^−1^⋅mol^−1^). The equilibrium constants (*K*) for the unfolding reaction of scPCNA and for the formation of the unfolding intermediate of hPCNA (in the range 0–2.5 M urea) can be expressed as:
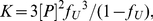
(7)where [*P*] is the protein concentration and *f_U_* is the fraction of unfolded protein, which can be calculated by the following expression:
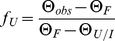
(8)


The values of *K* and Δ*G_U_* were calculated at each denaturant concentration and extrapolated to zero denaturant concentration according to the linear method [37]. The fitting of both scPCNA and hPCNA experimental curves was performed using the program Gnuplot (www.gnuplot.info). The experimental curves have been measured twice with essentially the same results.

### NMR measurements and analysis

The deposited ^13^C chemical shifts of PCNA come from perdeuterated molecules [11], [12]. In order to identify the secondary structure elements based on the comparison of these chemical shifts with tabulated values from protonated proteins a correction for the deuterium isotope effect was applied following empirical rules [39]. NOEs between backbone amide protons were identified in 4D ^15^N,^15^N-NOESY experiments recorded with a mixing time of 150 ms. The exchange of scPCNA and hPCNA amide protons for solvent deuterons was measured on a Bruker AV 700 spectrometer equipped with a z-gradient probe at 35°C. The decay in the ^1^H-^15^N correlations was measured with HSQC (hPCNA) or TROSY experiments (scPCNA) [40]. The proton-deuterium exchange was initiated by dissolving in D_2_O a previously lyophilized protein sample (scPCNA), or changing the protonated buffer into buffered D_2_O by several cycles of concentration by ultrafiltration and dilution (hPCNA). The values of the final sample volume and protein concentration were approximately 400 µL and 1 mM, respectively. The composition of the deuterated buffer was: 137 mM NaCl, 2.7 mM KCl, 10 mM sodium phosphate, 2 mM potassium phosphate pD 7.0 (pH meter reading, uncorrected for isotope effects). The first 2D ^1^H-^15^N correlation experiment was acquired within 1 hr from the initiation of the exchange. Successive experiments were acquired over a time period of 50 (scPCNA) or 3 days (hPCNA) at various intervals and with a larger number of scans in the later experiments to increase the signal-to-noise ratio (between 32 and 256). The same processing scheme was applied to all spectra and the peak intensities were scaled to account for the different number of scans. Hydrogen exchange rates were determined by fitting the decay in cross-peak intensities versus time to the single-exponential equation
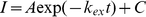
(9)using the rate analysis module of CCPNMR [41], where *I* represents the intensity of the cross-peak, *A* is the amplitude of the exchange curve, *k_ex_* is the observed exchange rate, *t* is the time after D_2_O buffer exchange and *C* is a constant that takes into account the residual non deuterated water. A *k_ex_* upper limit value for protons whose intensities were absent or very weak in the first experiment was estimated taking into account the time required for sample preparation and the duration of the first experiment. Assuming that remaining intensities of <10% could not be reliably measured, the fastest exchanging amide protons had a *k_ex_* of 1.3⋅10^−2^ min^−1^ and 2.2⋅10^−2^ min^−1^ in scPCNA and hPCNA, respectively. The protection factor of an amide proton (*PT*) is defined as the extent to which the measured exchange rate (*k_ex_*) is reduced relative to the exchange rate (*k_int_*) of a non-hydrogen bonded amide in an unstructured peptide with the same dipeptide sequence and at the same pH and temperature [23]. Thus *PT*  =  *k_ex_/k_int_*. The intrinsic exchange rate constants, *k_int_*, were calculated for every amino acid in the scPCNA sequence using the web tool available at www.fccc.edu/research/labs/roder/sphere [42].

### Comparison of 3D structures

Sequence alignment of the PCNA sequences was performed using the Uniprot Blast tool [32]. Secondary structures were assigned according to the annotations from the 3D structures in the corresponding PDB files (1PLQ for scPCNA, 1VYM for hPCNA, and 1GE8 for pfPCNA) [4], [5], [43]. The analysis of the intermonomer interfaces in PCNA (hydrogen bonds, ion pairs and accessible surface areas) was carried out using the PDB*e*PISA tool for exploration of macromolecular interfaces (http://www.ebi.ac.uk/msd-srv/prot_int/pistart.html).

### Supporting Information

Eight figures showing CD data, summary of NOEs delineating the β-sheet of PCNA molecules in solution, the assigned ^1^H-^15^N correlation NMR spectrum of scPCNA, the oligomerization analysis of PCNA by analytical ultracentrifugation and multiangle light scattering, three examples of the kinetics of scPCNA amide proton exchange with solvent deuterons, one table with the measured exchange rates and the calculated protection factors and one note on circular dichroism and solvent exchange. This material is available free of charge via the Internet at http://pubs.acs.org.

## Supporting Information

Figure S1 CD spectra of yeast and human PCNA. Far-UV CD spectra of scPCNA (51 μM) and hPCNA (54 μM) at 35°C in 20 mM sodium phosphate buffer pH 7.0, 150 mM NaCl, with and without 9 M urea or 7.4 M GuHCl.
(TIF)

Figure S2 Scheme of the regular secondary structure of each monomer of PCNA as seen in their X-Ray structures together with the β-sheet H_N_-H_N_ NOEs observed in the NMR spectra of (**A**) scPCNA and (**B**) hPCNA (the NOEs in coils and helices are not shown). The three β-sheets are arranged in a similar way as displayed in figure 2 of the report by Krishna et al. [4] and the approximate position of the helices are indicated with open circles. Each strand is labeled accordingly with the label at its N-terminus. These labels are the same as used in figure 1 of the current article.
(TIF)

Figure S3 Sedimentation velocity of scPCNA and hPCNA molecules recorded under different buffer, temperature and concentration.
(TIF)

Figure S4 Multiangle light scattering analysis of PCNA oligomerization. Gel filtration elution profiles as measured by UV absorvance at 280 nm (black trace) and molar mass (red trace) of (**A**) scPCNA and (**B**) hPCNA. The measured molar mass and fraction for each peak is indicated. Proteins were eluted at 3 or 0.3 mg/ml (∼ 100 or 10 μM) in 20 mM sodium phosphate, 150 mM NaCl, 0.03% (w/v) sodium azide, pH 7.0. MALS directs light from a 685-nm laser through a flow cell such that the intensity of light scattered by the sample is detected simultaneously at several scattering angles. Software provided by the manufacturer calculates the molecular weight of a species from the intensity of the scattered light according to Rayleigh light scattering principles. The intensity of scattered light is proportional to the molar mass, concentration of the solute and square of the *dn/dc* of the solute.
(TIF)

Figure S5 Thermal denaturation of PCNA. The CD signal of 17 μM (**A**) or 1.7 μM (**B**) scPCNA (thick line) and hPCNA (thin line) in 20 mM sodium phosphate, 150 mM NaCl, pH 7.0, is represented in MRE units as a function of temperature. The wavelength was set at 214 nm (scPCNA) or 217 nm (hPCNA), the minimum in the corresponding spectrum at room temperature.
(TIF)

Figure S6 Far-UV CD spectra of 17 μM scPCNA (**A**) or hPCNA (**B**) in 20 mM sodium phosphate, 150 mM NaCl, pH 7.0, recorded at different temperatures along the thermal denaturation curves corresponding to figure 3 in the manuscript.
(TIF)

Figure S7 ^1^H-^15^N TROSY spectrum of triply-labeled hPCNA with indication of assignments. Asn and Gln side chain NH_2_ correlations are boxed and appear distorted due to the optimization of the pulse sequence to select the TROSY component of the multiplet. Negative contour levels are plotted in red.
(TIF)

Figure S8 Examples of fittings of the scPCNA TROSY ^1^H-^15^N cross-peak intensities as a function of time after D_2_O buffer exchange. (**A**) ^180^Val, k_ex_  =  1.3 ± 0.2 · 10^−3^ min^−1^; (**B**) ^70^Met, k_ex_  =  8.6 ± 0.9 · 10^−4^ min^−1^; (**C**) ^34^Gly, k_ex_  =  2.9 ± 0.5 · 10^−3^ min^−1^.
(TIF)

Table S1 (DOC)

Note S1 On CD and solvent exchange of amide protons.
(DOC)
