# Correction: Reduced Stability and Increased Dynamics in the Human Proliferating Cell Nuclear Antigen (PCNA) Relative to the Yeast Homolog

**DOI:** 10.1371/journal.pone.0097057

**Published:** 2014-04-30

**Authors:** The PLOS ONE Staff

There is an error in Figure 2: The lower-left panel should labeled as "B" and the upper-right panel should be labeled as "C".

Equations 1–5 are formatted incorrectly. The publisher apologizes for the errors. Please see the correct equations here.
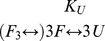
(1)

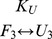
(2)

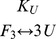
(3)

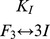
(4)

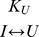
(5)

