# Supplementary figures and images for: Reduced Stability and Increased Dynamics in the Human Proliferating Cell Nuclear Antigen (PCNA) Relative to the Yeast Homolog
Source: PLoS One. 2011 Feb 18;6(2):e16600. doi: 10.1371/journal.pone.0016600 (PMC3041752; doi:10.1371/journal.pone.0016600)

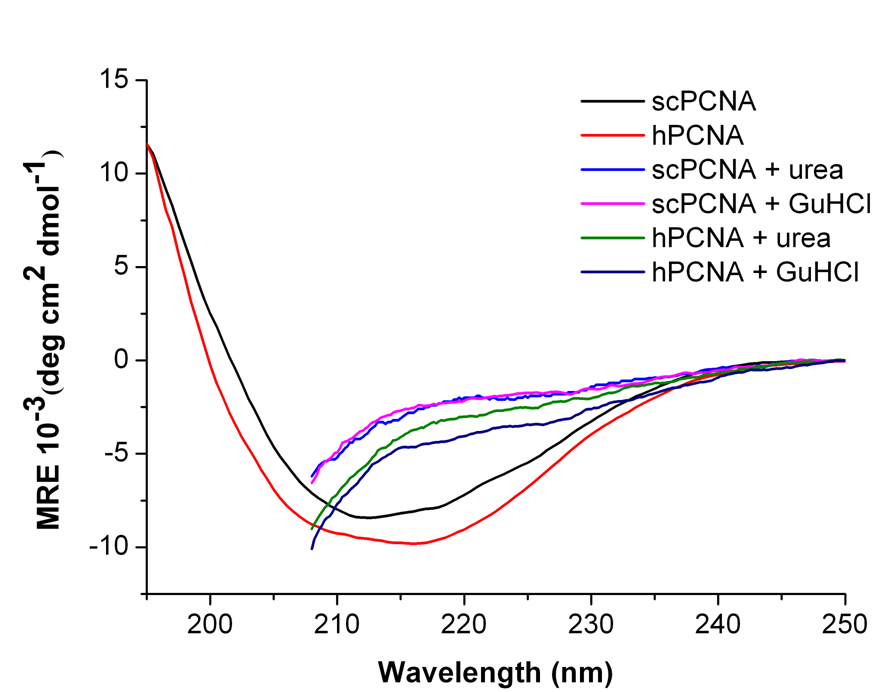

Supplement: Figure S1 — CD spectra of yeast and human PCNA. Far-UV CD spectra of scPCNA (51 μM) and hPCNA (54 μM) at 35°C in 20 mM sodium phosphate buffer pH 7.0, 150 mM NaCl, with and without 9 M urea or 7.4 M GuHCl. (TIF) [file pone.0016600.s001.tif]

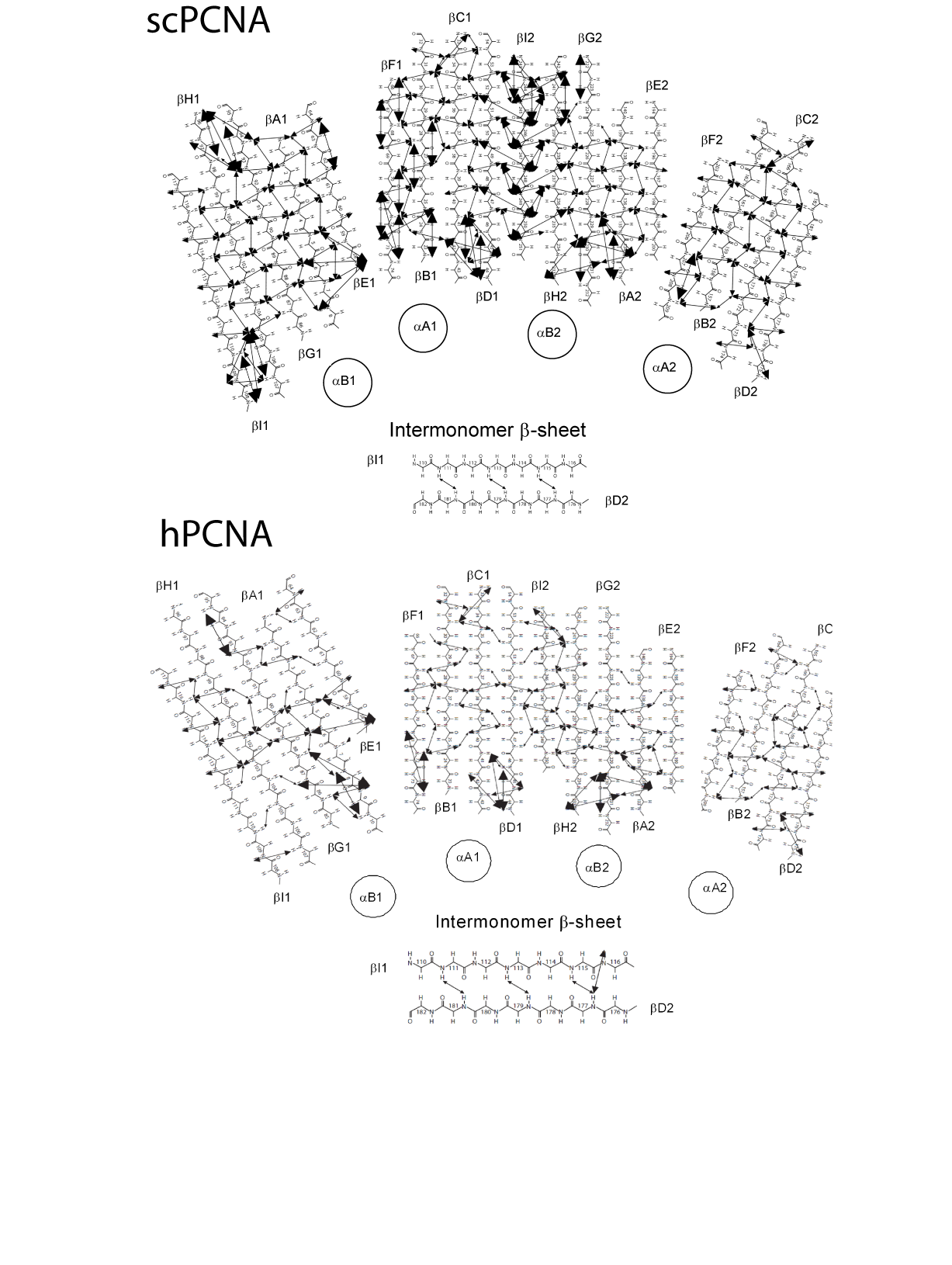

Supplement: Figure S2 — Scheme of the regular secondary structure of each monomer of PCNA as seen in their X-Ray structures together with the β-sheet HN-HN NOEs observed in the NMR spectra of (A) scPCNA and (B) hPCNA (the NOEs in coils and helices are not shown). The three β-sheets are arranged in a similar way as displayed in figure 2 of the report by Krishna et al. [4] and the approximate position of the helices are indicated with open circles. Each strand is labeled accordingly with the label at its N-terminus. These labels are the same as used in figure 1 of the current article. (TIF) [file pone.0016600.s002.tif]

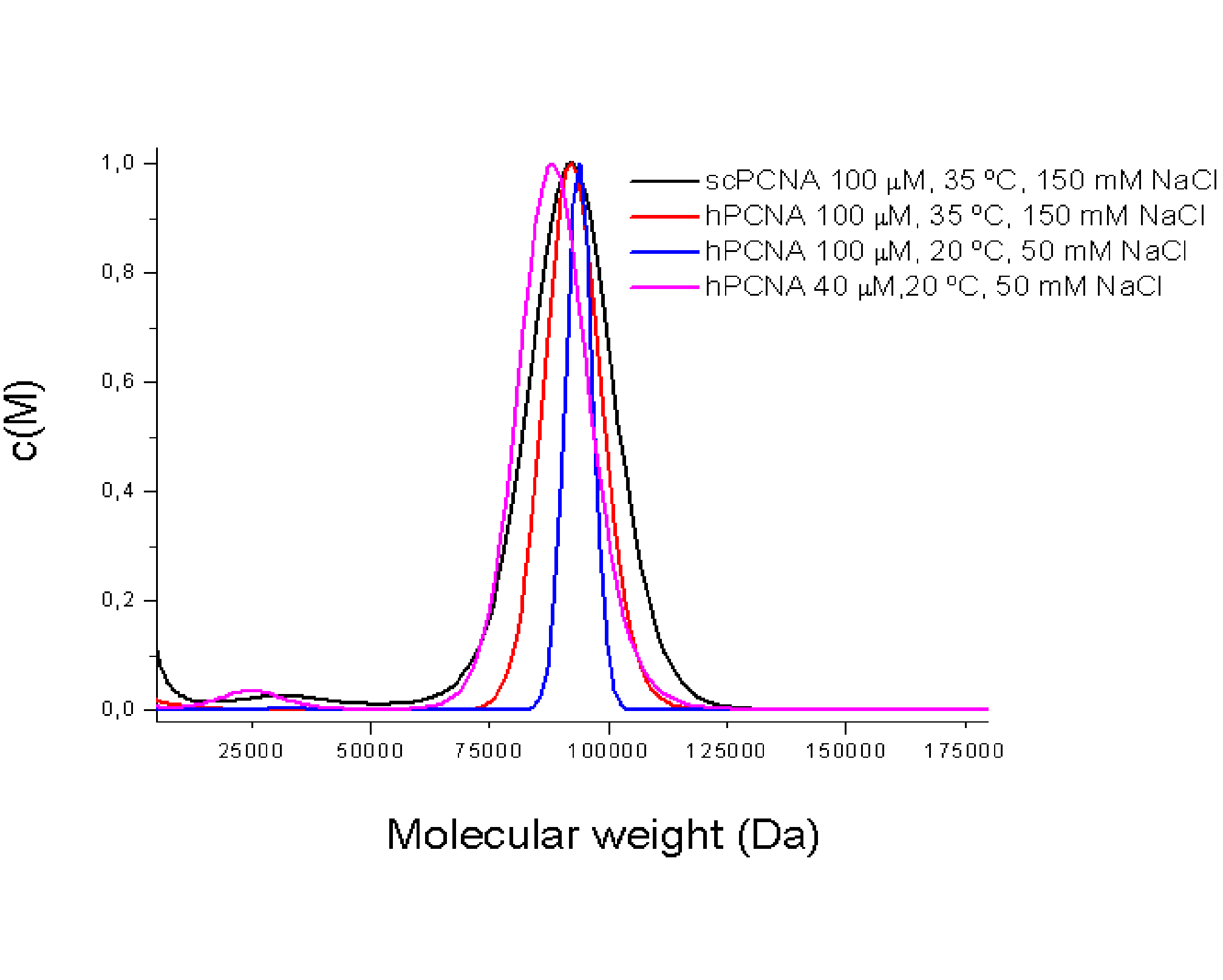

Supplement: Figure S3 — Sedimentation velocity of scPCNA and hPCNA molecules recorded under different buffer, temperature and concentration. (TIF) [file pone.0016600.s003.tif]

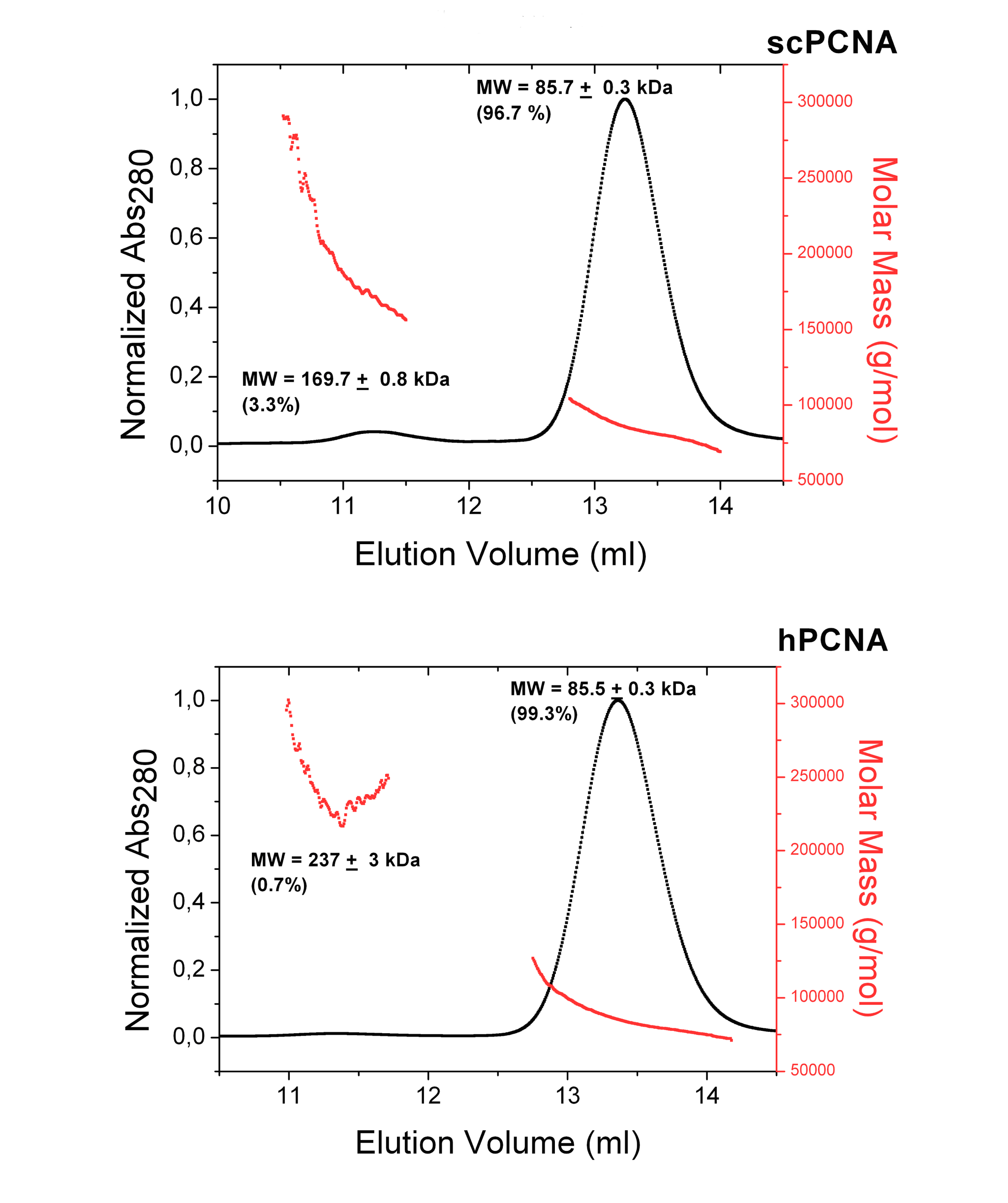

Supplement: Figure S4 — Multiangle light scattering analysis of PCNA oligomerization. Gel filtration elution profiles as measured by UV absorvance at 280 nm (black trace) and molar mass (red trace) of (A) scPCNA and (B) hPCNA. The measured molar mass and fraction for each peak is indicated. Proteins were eluted at 3 or 0.3 mg/ml (∼ 100 or 10 μM) in 20 mM sodium phosphate, 150 mM NaCl, 0.03% (w/v) sodium azide, pH 7.0. MALS directs light from a 685-nm laser through a flow cell such that the intensity of light scattered by the sample is detected simultaneously at several scattering angles. Software provided by the manufacturer calculates the molecular weight of a species from the intensity of the scattered light according to Rayleigh light scattering principles. The intensity of scattered light is proportional to the molar mass, concentration of the solute and square of the dn/dc of the solute. (TIF) [file pone.0016600.s004.tif]

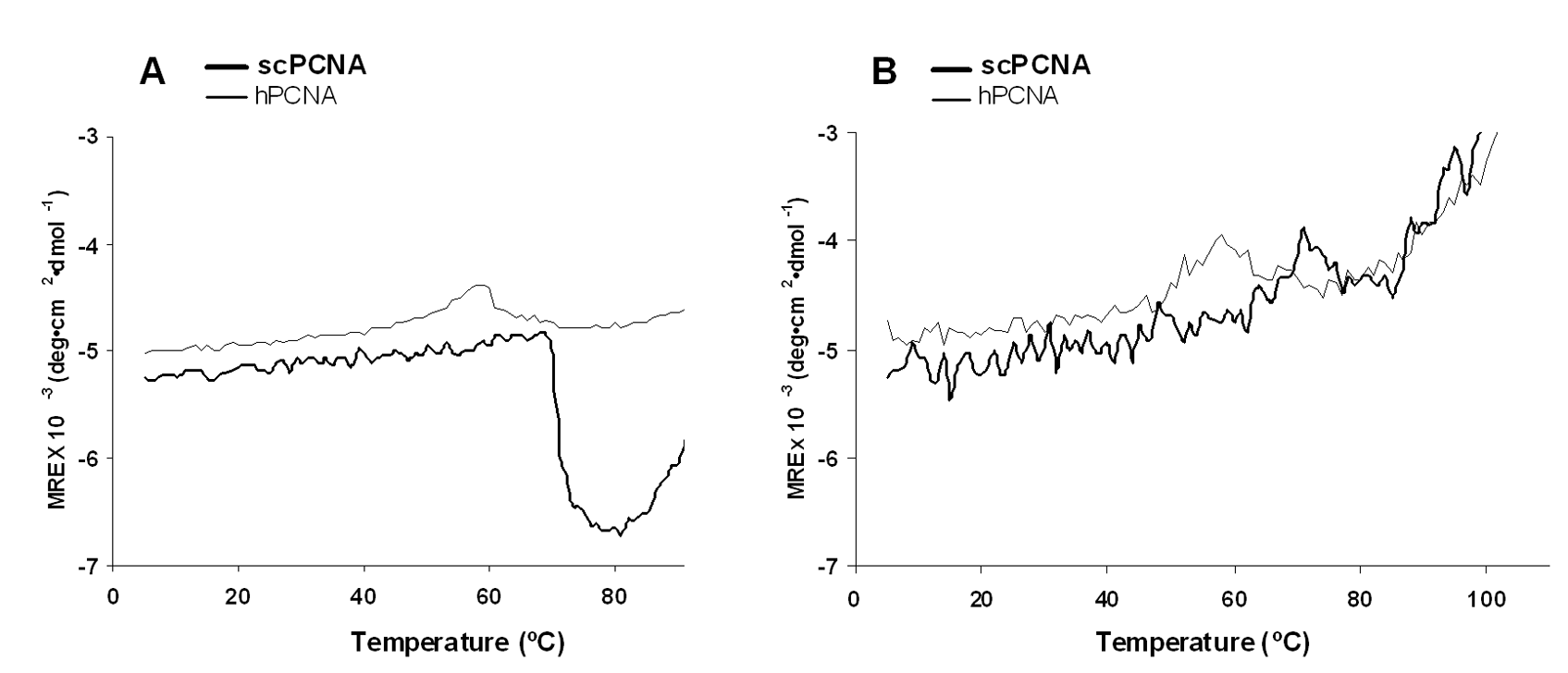

Supplement: Figure S5 — Thermal denaturation of PCNA. The CD signal of 17 μM (A) or 1.7 μM (B) scPCNA (thick line) and hPCNA (thin line) in 20 mM sodium phosphate, 150 mM NaCl, pH 7.0, is represented in MRE units as a function of temperature. The wavelength was set at 214 nm (scPCNA) or 217 nm (hPCNA), the minimum in the corresponding spectrum at room temperature. (TIF) [file pone.0016600.s005.tif]

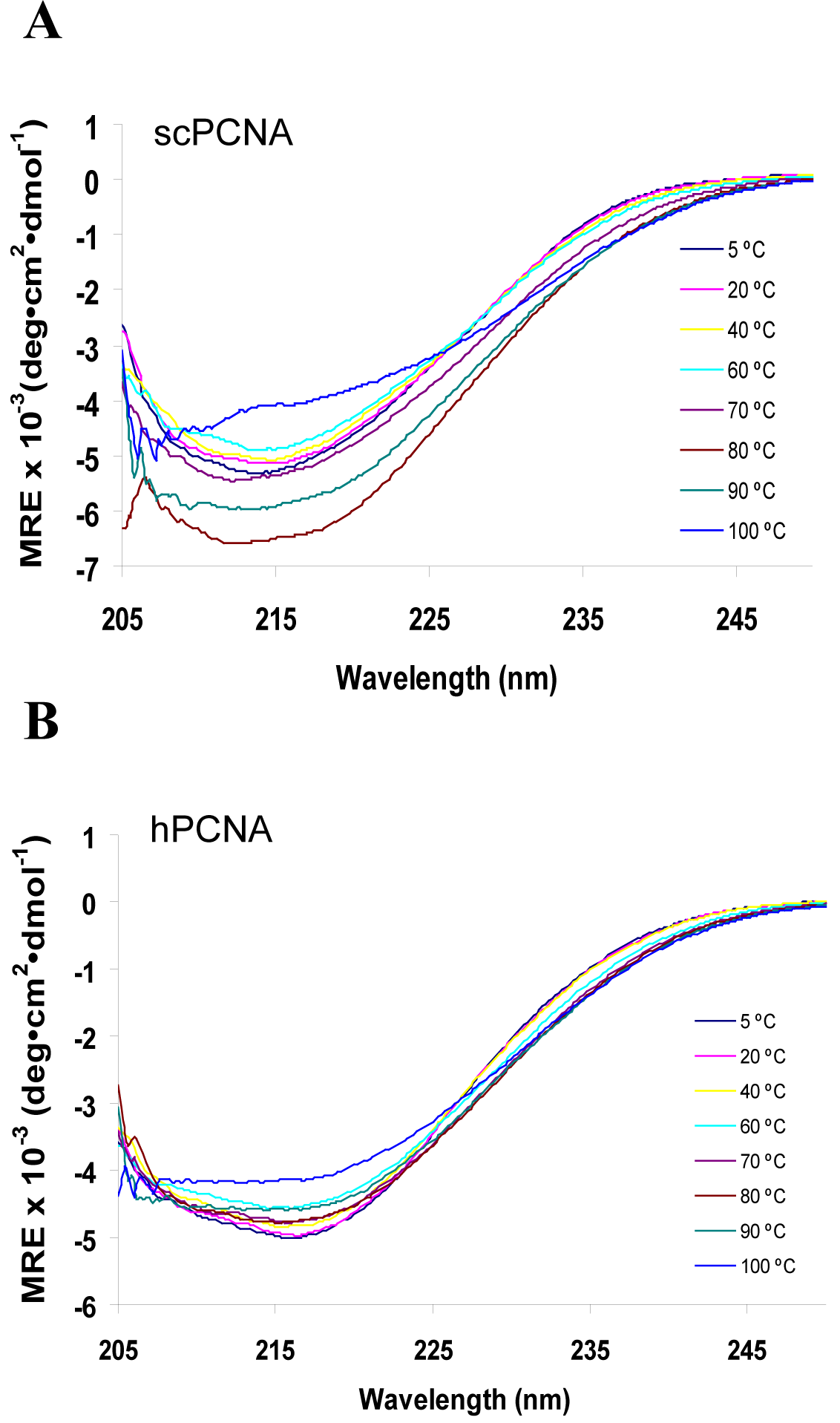

Supplement: Figure S6 — Far-UV CD spectra of 17 μM scPCNA (A) or hPCNA (B) in 20 mM sodium phosphate, 150 mM NaCl, pH 7.0, recorded at different temperatures along the thermal denaturation curves corresponding to figure 3 in the manuscript. (TIF) [file pone.0016600.s006.tif]

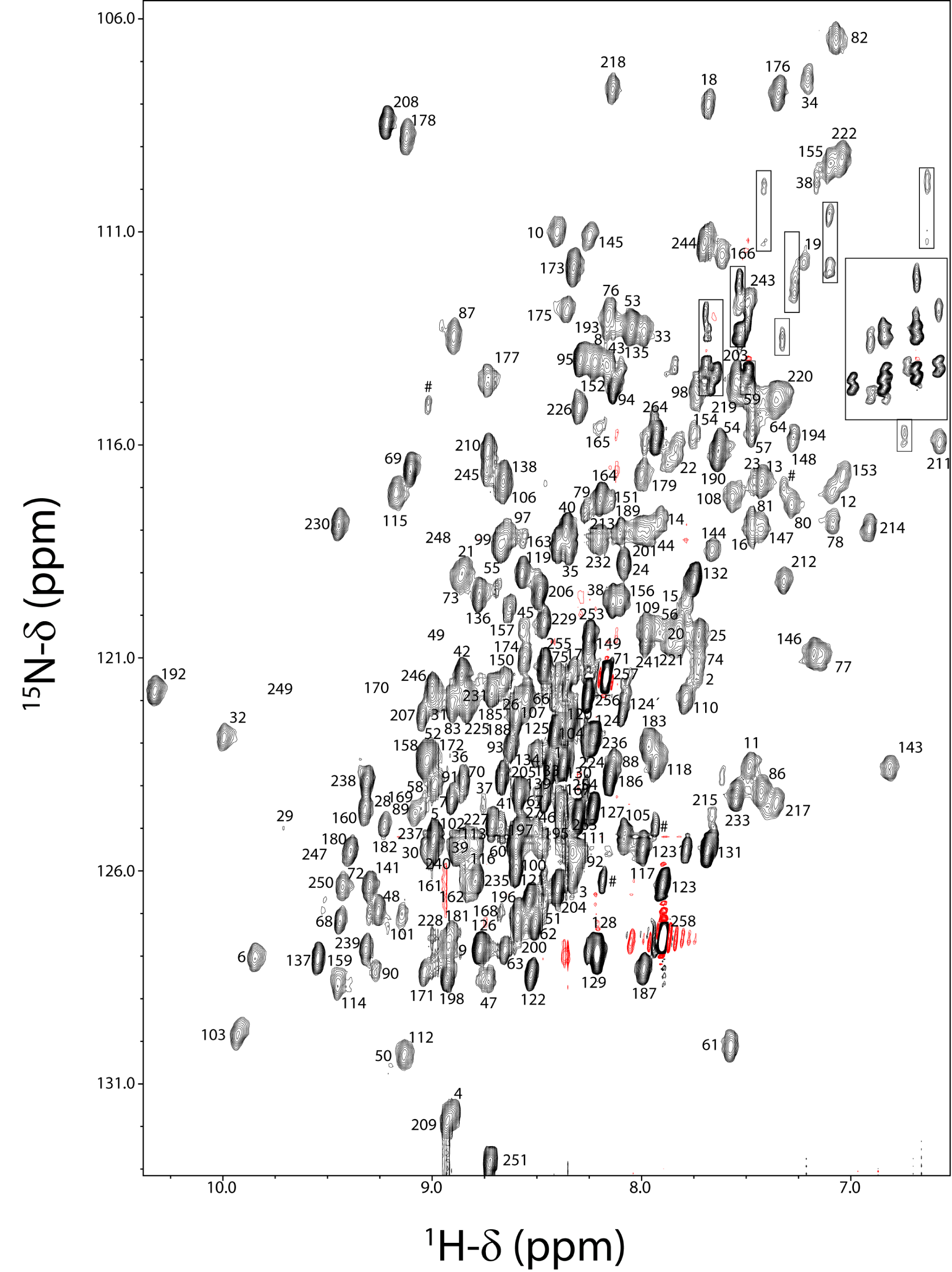

Supplement: Figure S7 — 1H-15N TROSY spectrum of triply-labeled hPCNA with indication of assignments. Asn and Gln side chain NH2 correlations are boxed and appear distorted due to the optimization of the pulse sequence to select the TROSY component of the multiplet. Negative contour levels are plotted in red. (TIF) [file pone.0016600.s007.tif]

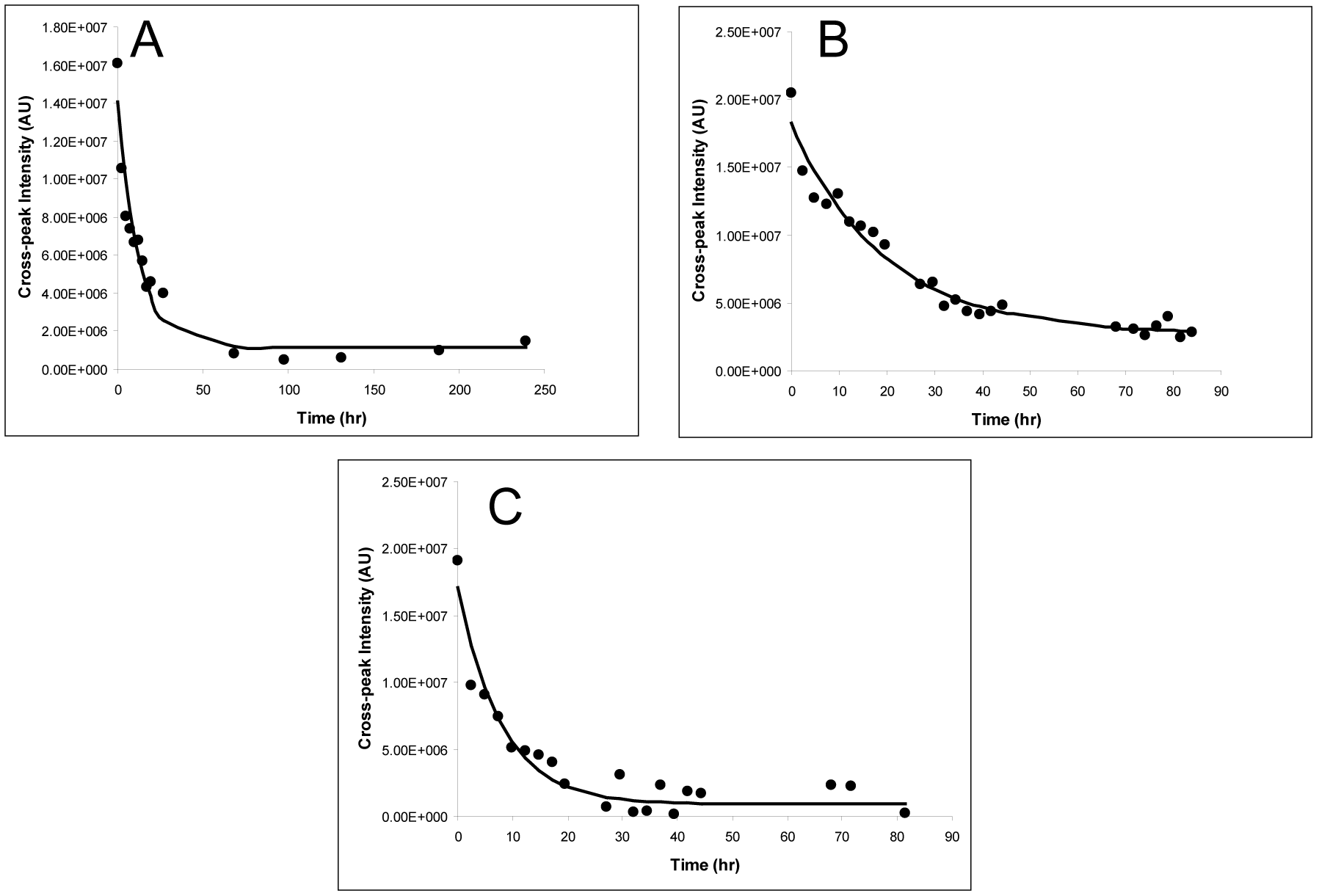

Supplement: Figure S8 — Examples of fittings of the scPCNA TROSY 1H-15N cross-peak intensities as a function of time after D2O buffer exchange. (A) 180Val, kex = 1.3 ± 0.2 · 10−3 min−1; (B) 70Met, kex = 8.6 ± 0.9 · 10−4 min−1; (C) 34Gly, kex = 2.9 ± 0.5 · 10−3 min−1. (TIF) [file pone.0016600.s008.tif]
