# Supplementary material for: Reduced Stability and Increased Dynamics in the Human Proliferating Cell Nuclear Antigen (PCNA) Relative to the Yeast Homolog
Source: PLoS One. 2011 Feb 18;6(2):e16600. doi: 10.1371/journal.pone.0016600 (PMC3041752; doi:10.1371/journal.pone.0016600)
Supplement: Table S1 — (DOC) [file pone.0016600.s009.doc]

**Supporting Table S1. Intrinsic (k_int_) and measured (k_ex_) exchange rate constants and protection factors calculated for the scPCNA backbone amides at pH 7.0 and 35 ºC. The fitting errors for the k_ex_ and the protection factors are shown.**

| Residue | k_ex_ (x10^4^ min^-1^) | k_int_ (x10^-2^ min^-1^) | -ln(k_ex_/k_int_) |
| --- | --- | --- | --- |
| 29 | 2.3 ± 0.8 | 13.44 | 15.6 ± 0.3 |
| 34 | 29.4 ± 4.6 | 12.6 | 13.0 ± 0.1 |
| 35 | 9.0 ± 1.3 | 2.814 | 12.7 ± 0.2 |
| 36 | 8.9 ± 2.4 | 1.122 | 11.7 ± 0.3 |
| 47 | 4.6 ± 0.5 | 1.656 | 12.8 ± 0.1 |
| 48 | 9.7 ± 1.4 | 1.254 | 11.8 ± 0.1 |
| 49 | 6.9 ± 1.2 | 17.34 | 14.7 ± 0.2 |
| 62 | 30.8 ± 3.5 | 60 | 14.5 ± 0.1 |
| 68 | 1.9 ± 0.2 | 4.254 | 14.6 ± 0.1 |
| 70 | 8.6 ± 0.9 | 14.76 | 14.4 ± 0.1 |
| 78 | 29.2 ± 6.2 | 2.508 | 11.4 ± 0.2 |
| 79 | 53.9 ± 10.5 | 1.584 | 10.3 ± 0.2 |
| 89 | 1.4 ± 0.1 | 5.358 | 15.2 ± 0.1 |
| 90 | 1.1 ± 0.1 | 4.254 | 15.2 ± 0.1 |
| 101 | 2.6 ± 0.6 | 1.584 | 13.3 ± 0.2 |
| 103 | 7.2 ± 2.0 | 3.624 | 13.1 ± 0.3 |
| 115 | 71.0 ± 7.5 | 26.82 | 12.8 ± 0.1 |
| 139 | 7.6 ± 2.0 | 5.358 | 13.5 ± 0.3 |
| 149 | 1.5 ± 0.1 | 8.88 | 15.6 ± 0.1 |
| 161 | 0.7 ± 0.2 | 5.874 | 16.0 ± 0.2 |
| 164 | 0.8 ± 0.1 | 14.76 | 16.8 ± 0.2 |
| 168 | 13.1 ± 4.6 | 5.484 | 12.9 ± 0.4 |
| 169 | 2.7 ± 0.6 | 7.74 | 14.9 ± 0.2 |
| 170 | 1.7 ± 1.1 | 2.34 | 14.1 ± 0.6 |
| 171 | 20.1 ± 4.4 | 7.38 | 12.8 ± 0.2 |
| 180 | 13.2 ± 2.2 | 4.062 | 12.6 ± 0.1 |
| 182 | 1.3 ± 0.1 | 1.122 | 13.7 ± 0.1 |
| 205 | 18.5 ± 3.2 | 1.776 | 11.5 ± 0.2 |
| 225 | 1.5 ± 0.1 | 3.378 | 14.6 ± 0.1 |
| 226 | 8.2 ± 1.4 | 13.8 | 14.3 ± 0.2 |
| 228 | 13.9 ± 0.7 | 7.2 | 13.2 ± 0.3 |
| 238 | 10.2 ± 3.4 | 13.44 | 14.1 ± 0.3 |
| 247 | 10.2 ± 2.4 | 7.2 | 13.5 ± 0.2 |
| 248 | 8.9 ± 2.5 | 9.3 | 13.9 ± 0.3 |
| 249 | 10.2 ± 2.6 | 6.72 | 13.4 ± 0.3 |
