# Supplementary material for: Reduced Stability and Increased Dynamics in the Human Proliferating Cell Nuclear Antigen (PCNA) Relative to the Yeast Homolog
Source: PLoS One. 2011 Feb 18;6(2):e16600. doi: 10.1371/journal.pone.0016600 (PMC3041752; doi:10.1371/journal.pone.0016600)
Supplement: Note S1 — On CD and solvent exchange of amide protons. (DOC) [file pone.0016600.s010.doc]

**Supporting Note on H-D exchange**

According to the Linderstrøm-Lang model, the exchange reaction of a protected amide proton takes place when it is transiently exposed to the solvent as a result of a structural fluctuation ^1^. There are two main limiting mechanisms for exchange, named EX1 and EX2. In the EX1 limit, each fluctuation to the open, exchange competent conformation leads to exchange and the measured *k_ex_* equals the constant rate of the opening process. In the EX2 mechanism, which applies to proteins under most conditions ^2^, each conformational fluctuation does not necessarily lead to the exchange of the exposed amide protons; exchange itself is the limiting step of the reaction. Thus, in the EX2 limit the backbone amide proton exchange with the solvent provides a window for observing main chain dynamics at time scales similar to, or faster than, the time scale of the intrinsic amide proton exchange (1-10^2^ s^-1^ for scPCNA at these experimental conditions). The intrinsic exchange corresponds to the unfolded polypeptide chain and is conformation independent at a given pH and temperature, but it is amino acid and sequence dependent. Irrespective of which mechanism governs the exchange reaction.

Since the hydrogen-exchange assay provides insight into the presence of locally unfolded conformations under native conditions in exchange with the folded ones, smaller exchange rates correspond to slower local backbone dynamics^2; 3; 4; 5^.

**Supporting Note on Circular Dichroism data**

The far-UV circular dichroism spectra of scPCNA and hPCNA are consistent with proteins with mixed α/β/coil secondary structures with no predominant CD signal from any of them (Figure S1). However, although the two homologs have virtually identical crystal structures, there are differences in their CD spectra. At wavelengths higher than 200 nm hPCNA displays lower mean residue molar ellipticity values than scPCNA, and the wavelength at minimum is 4 nm shorter. These differences could be due to a different global secondary structure composition for each protein in solution at 35 °C. However thet NMR data show that the same secondary structure elements (helices and β-strands) as well as β-sheets exist in both proteins. This analysis is however restricted to the regular secondary structure elements, therefore it may be that different structures at the coil regions in the two proteins in solution account for the differences in their CD spectra.

The differences in the CD spectra of the two proteins are likely due to the 17 phenylalanine residues in scPCNA (only 8 in hPCNA): aromatic residues can have a non-negligible contribution to the CD spectrum in the far UV, and it has been previously quantified for the case of phenylalanine residues in helical peptides ^6^.

The thermal denaturation of the two homologs followed by the changes in their CD signal at 222 nm as a function of temperature show significant differences (Fig. S5). The denaturation of scPCNA starts at about 70 ºC, whereas hPCNA reaches a partially unfolded state already at 60 ºC. The denaturation of scPCNA begins with a dramatic decrease of the ellipticity, reflecting a conformational change likely due to the conversion of some β-sheet and/or coil regions of the protein into non-native helical structure. Above 80 ºC the ellipticity increases rapidly with temperature indicating a loss of secondary structure. In agreement with this observation, the CD spectrum of scPCNA at 100 ºC indicates an increase in random coil content with respect to lower temperatures (Figure S6). The thermal denaturation of hPCNA shows a biphasic behavior of thermal unfolding, with two transitions of low cooperativity (Figure S6A). The first transition has a mid point temperature of approximately 55 ºC, the ellipticity reaches a maximum at 60 ºC, and then decreases with a shallow minimum at about 80 ºC. The shape of the curve is similar to the denaturation of scPCNA, but the amount of non-native helical structure acquired in the 60-80 ºC region is much smaller (also seen in the spectra at different temperatures), and the transitions are less cooperative. Although no protein precipitation is observed in the yeast and human PCNA samples, the thermal denaturations appear to be irreversible since the CD spectra are not recovered after cooling the samples down to 35 °C (the one dimensional ^1^H-NMR spectra of these heated and cooled samples differ also from the native protein spectra and show non-dispersed signals, data not shown).

The thermal denaturation on samples at ten-fold lower protein concentrations (Fig. 6B) shows almost the same curve for hPCNA (within the experimental error, which is larger for the low concentration measurements), while scPCNA experiences only small changes in secondary structure at the same temperature values where the transitions in the more concentrated sample were observed. Furthermore, the curves for the two proteins are remarkably similar except for a shift of about 15 °C to larger temperatures in the case of sPCNA.

The thermal denaturation of both PCNA molecules is complex and difficult to interpret. At high concentrations the processes are irreversible and no quantitative analysis can be done on the relative stability at physiological temperature. For scPCNA at 17 μM the ellipticity changes and the spectra at different temperatures (Figure S5) suggest that the thermal denaturation drives the formation of non-native α-helical structures, possibly stabilized by intermolecular hydrophobic interactions, which are favored at high temperatures. At 1.7 μM the melting curves of the two proteins are very similar but shifted by about 15 ºC in scPCNA relative to hPCNA. Therefore scPCNA is more resistant than scPCNA to thermal denaturation.

**Supporting Methods**

*Far-UV CD spectroscopy.* Spectra were recorded in the range 250-200 nm with a Jasco-815 polarimeter using quartz cuvettes of 1.0, 0.2 or 0.01 cm path lengths. Thermal denaturation was performed by recording the CD signal of scPCNA (at a monomer concentration of 17.0 or 1.7 μM, λ = 214 nm) or hPCNA (14.0 or 1.4 μM , λ = 217 nm) in 20 mM sodium phosphate buffer, 150 mM NaCl, pH 7.0, from 5 to 105 ºC with 1 ºC increments, at a rate of 1 ºC/min.

*Analytical ultracentrifugation*

Sedimentation velocity studies were carried out in a Beckman Optima XL-A analytical ultracentrifuge equipped with UV/visible optics, an An50Ti rotor, with 0.3 cm double sector centerpieces of Epon charcoal. Measurements were made on Gadd45α proteins at different concentrations in 300 µL of 20 mM sodium phosphate, pH 7.0 and 50 or 150 mM NaCl. The sedimentation velocity experiments were carried out at 42,000 rpm over five hours and absorbance scans were taken at 280 nm with 40 and 100 μM samples of the PCNA molecules. Since scPCNA does not have trytophane reasidues and absorbs less light than hPCNA, the measurements at small concentrations would have much higher errors and were not obtained. Sedimentation coefficients were calculated using the continuous distribution c(s) Lamm equation model as implemented in the SEDFIT program ^7^. These experimental sedimentation coefficient values were corrected to standard conditions in order to obtain the corresponding S_20,w_ values using the SEDNTERP program ^8^.

*Size Exclusion Chromatography and Multi Angle Light Scattering*

scPCNA and hPCNA masses were measured using a multiangle light scattering (MALS) instrument (DAWN Heleos II, Wyatt Technology) at room temperature. The instrument was placed in line with a refractive index detector (Optilab Rex, Wyatt Technologies) during size-exclusion chromatography (SEC). SEC was performed on a Äkta Explorer FPLC Instrument with a Superdex 200 10/300 GL column (GE Healthacare) equilibrated in buffer containing 20 mM sodium phosphate, 150 mM NaCl, 0.03 % (w/v) sodium azide, pH 7.0, and with a flow rate of 0.5 ml/min. Protein stock solutions were diluted in the equilibration buffer at a concentration of 3 mg/ml, filtered and 120 μl were applied.

**Supporting information references**

1. Linderstrøm-Lang, K. U. (1955). Deuterium exchange between peptides and water. *Chem. Soc. Spec. Publ.* 2, 1-20.

2. Dempsey. (2001). Hydrogen Exchange in Peptides and Proteins Using NMR spectroscopy. *Prog Nucl Magn Reson Spectrosc* 39, 135-170.

3. Pantoja-Uceda, D., Arolas, J. L., Garcia, P., Lopez-Hernandez, E., Padro, D., Aviles, F. X. & Blanco, F. J. (2008). The NMR structure and dynamics of the two-domain tick carboxypeptidase inhibitor reveal flexibility in its free form and stiffness upon binding to human carboxypeptidase B. *Biochemistry* 47, 7066-78.

4. Hernandez, G., Jenney, F. E., Jr., Adams, M. W. & LeMaster, D. M. (2000). Millisecond time scale conformational flexibility in a hyperthermophile protein at ambient temperature. *Proc Natl Acad Sci U S A* 97, 3166-70.

5. Ishima, R. & Torchia, D. A. (2000). Protein dynamics from NMR. *Nat Struct Biol* 7, 740-3.

6. Viguera, A. R. & Serrano, L. (1995). Side-chain interactions between sulfur-containing amino acids and phenylalanine in alpha-helices. *Biochemistry* 34, 8771-9.

7. Schuck, P. (2000). Size-distribution analysis of macromolecules by sedimentation velocity ultracentrifugation and lamm equation modeling. *Biophys J* 78, 1606-19.

8. Laue, T. M., Shah, B. D., Ridgeway, T. M. & Pelletier, S. L. (1992). *Computer-aided interpretation of analytical sedimentation data for proteins.* Analytical Ultracentrifugation in Biochemistry and Polymer Science (Harding, S. E., Rowe, A. J. & Horton, J. C., Eds.), Royal Society of Chemistry, Cambridge, UK.

9. Krishna, T. S., Kong, X. P., Gary, S., Burgers, P. M. & Kuriyan, J. (1994). Crystal structure of the eukaryotic DNA polymerase processivity factor PCNA. *Cell* 79, 1233-43.
